# An artificial intelligence-based first-line defence against COVID-19: digitally screening citizens for risks via a chatbot

**DOI:** 10.1038/s41598-020-75912-x

**Published:** 2020-11-04

**Authors:** Alistair Martin, Jama Nateqi, Stefanie Gruarin, Nicolas Munsch, Isselmou Abdarahmane, Marc Zobel, Bernhard Knapp

**Affiliations:** 1 Data Science Department, Symptoma, Vienna, Austria; 2 Medical Department, Symptoma, Attersee, Austria; 3 Department of Internal Medicine, Paracelsus Medical University, Salzburg, Austria

**Keywords:** Health care, Signs and symptoms

## Abstract

To combat the pandemic of the coronavirus disease 2019 (COVID-19), numerous governments have established phone hotlines to prescreen potential cases. These hotlines have struggled with the volume of callers, leading to wait times of hours or, even, an inability to contact health authorities. Symptoma is a symptom-to-disease digital health assistant that can differentiate more than 20,000 diseases with an accuracy of more than 90%. We tested the accuracy of Symptoma to identify COVID-19 using a set of diverse clinical cases combined with case reports of COVID-19. We showed that Symptoma can accurately distinguish COVID-19 in 96.32% of clinical cases. When considering only COVID-19 symptoms and risk factors, Symptoma identified 100% of those infected when presented with only three signs. Lastly, we showed that Symptoma’s accuracy far exceeds that of simple “yes–no” questionnaires widely available online. In summary, Symptoma provides unparalleled accuracy in systematically identifying cases of COVID-19 while also considering over 20,000 other diseases. Furthermore, Symptoma allows free text input, furthered with disease-specific follow up questions, in 36 languages. Combined, these results and accessibility give Symptoma the potential to be a key tool in the global fight against COVID-19. The Symptoma predictor is freely available online at https://www.symptoma.com.

## Introduction

Currently, the world is facing an unprecedented health crisis caused by the novel coronavirus disease 2019 (COVID-19). To track and hopefully curb this pandemic, large-scale laboratory testing for COVID-19 is becoming widespread. However, capacities are far from being able to test whole populations. Therefore many countries have established phone hotlines to pre-screen persons who are unsure about their COVID-19 infection status. Only after talking to an operator and being identified as a potential case, often due to being symptomatic, will laboratory testing occur. However, these hotlines are severely overrun worldwide, leading to hour-long waiting periods and, even, disconnected lines. This ultimately leads to many COVID-19 cases going undiagnosed.

One solution to the overwhelming number of calls inundating hotlines is to pre-screen them using computer-based approaches. Digital services are already utilised for assessment and triage in various countries to reduce stress on the emergency responders, e.g., the “digital 111” service provided by the NHS in England^1,2^. These methods can be grouped into two categories. Firstly, a large number of simple yes/no online questionnaires are available. These questionnaires lead straight to the point but are limited in their informative value. They do not provide a deep understanding of a patient’s health situation, they do not allow for the consideration of additional symptoms, they do not allow the generation of additional data for analysis, and, critically, they are often language- and/or country-specific.

The alternative to these questionnaires is general-purpose symptom checkers which allow a user to list or select their symptoms before being provided with potential causes. Several have already been developed over recent years (benchmarked in Nateqi et al.)^3^. However, most of these symptom checkers are highly restricted in the number of diseases taken into account as building up the underlying databases is fundamentally cost-intensive and slow. Furthermore, language ambiguities, such as synonyms, are hard to overcome, e.g., dyspnea is the medical term for shortness of breath, though is rarely used outside of the medical profession. This inevitably leads to small disease databases where users can only choose from a limited list of pre-defined symptoms, lowering their viability when a new disease emerges.

Recently, we showed that Symptoma, a symptom-to-disease digital health assistant, significantly outperforms other symptom checkers in the diagnoses of ear, nose, and throat (ENT) diseases^3^. In the following, we present the accuracy of Symptoma with regards to systematically identifying cases of COVID-19, both while considering over 20,000 other diseases and when only considering common misdiagnoses.

## Results

### Sensitivity and specificity

We consider a patient as COVID-19 positive if their symptoms cause COVID-19 to be returned in the first 30 diagnoses listed. COVID-19 returned below this, or not at all, results in a patient being classified as COVID-19 negative. This method of evaluation reflects that used in the most comprehensive review of symptom checkers to date as well as our prior publication^2,3^. It provides an evaluation of accuracy with regards to COVID-19 under the criteria used for the assessment of general diagnoses. Under this definition, Symptoma classifies nearly all 30 COVID-19 case descriptions correctly as COVID-19 cases (96.6% sensitivity), failing only when presented with a case containing only a single defining symptom of COVID-19 (**Fever**, Fatigue, Dizziness, Constipation, Rhonchi, Tachypnea, and Bilateral pneumonia). However, achieving 100% sensitivity is easy, e.g., by constructing a test that simply classifies every case as COVID-19.

To address this issue we also tested how well Symptoma performs on cases of non-COVID-19 patients. For this purpose, we use a set of 1112 British Medical Journal (BMJ) cases that stretch over 84 fields of medicine (see in “Methods” section). Of these 1112 cases, only 41 are classified as potential COVID-19 cases by Symptoma, with only seven of these ranking COVID-19 higher than the correct diagnoses. These seven cases relate to diseases that present similarly to COVID-19, however, have far lower incidence rates and, therefore, are deemed less likely, e.g. Severe Acute Respiratory Syndrome (SARS-CoV) or the Avian influenza A (H5N1) virus infection (bird flu). The results are summarized in Table 1.

**Table 1:** Sensitivity and specificity of Symptoma in COVID-19 cases and BMJ negative controls.

	Flagged as COVID-19 Risk	Not flagged as COVID-19 Risk
COVID-19 cases (n=30)	29 (TP)	1 (FN)
BMJ cases (n=1112)	41 (FP)	1071 (TN)
Sensitivity	96.66% (29 of 30 detected)	
Specificity	96.31% (41 of 1112 incorrectly detected)	
Accuracy	96.32% (1100 of 1142 predictions correct)	

Many of the control cases within the above test set may not be mistaken for COVID-19. As such, we repeated this analysis considering only those cases with symptoms commonly associated with COVID-19, e.g., fever or dry cough. Full details of this subset are given in the Methods. Under this constraint, 374 out of the 1112 BMJ cases were still considered for a total of 404 total cases (BMJ and COVID-19 cases). On this subset, Symptoma achieves a sensitivity, specificity and accuracy of 0.966, 0.901 and 90.6% respectively.

### Discovery speed and sensitivity

Identifying patients presenting with COVID-19 both quickly and efficiently is of utmost importance to digital risk classification. However, achieving both speed and accuracy simultaneously is remarkably difficult. Short, and therefore quick, questionnaires will typically have low specificity, while conversely, long questionnaires lack efficiency and speed, often containing many questions not pertinent to any given patient. Symptoma’s free text search allows quick, efficient, and complex queries of symptom’s without constraint to a predefined list of symptoms.

**Figure 1:**
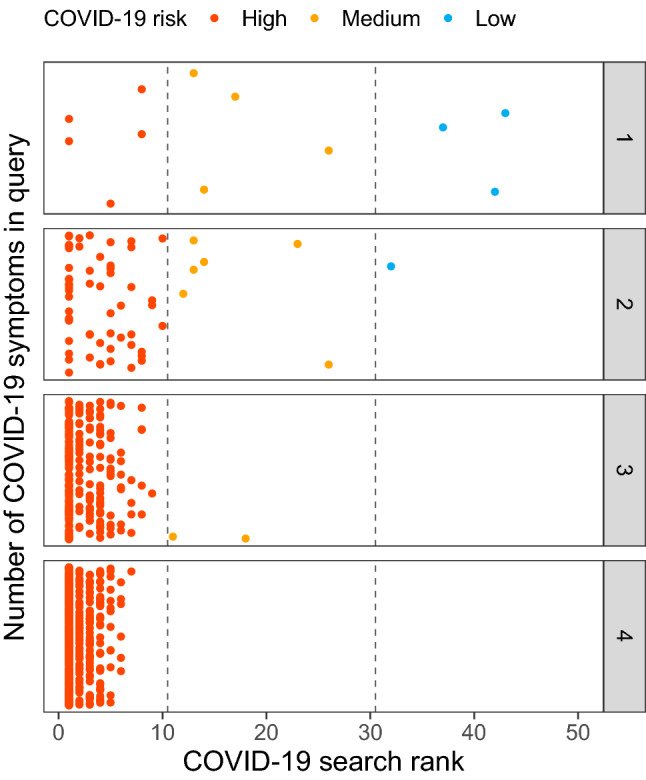
Identification of COVID-19 cases with regards to the number of query terms entered. On the x-axis, the search rank of the query in Symptoma is given against the y-axis where each panel considers a different number of symptoms in the query. All combinations of the reported COVID-19 symptoms are considered with each dot representing one unique combination. Points are jittered vertically for clarity only.

To highlight this with regards to COVID-19, we show in Fig. 1, the search rank of queries containing various numbers of symptoms known to be present in those infected with COVID-19 (see in “Methods” section). Key symptoms, such as suffering a fever or dry cough, leads to COVID-19 suggested within the top 30 search results immediately. Living in an area with a high incidence of COVID-19 or contact with a known case of COVID-19 results in a high-risk assessment immediately. The top 30 threshold is passed by 75%, 98.5%, and 100% of one, two, and three symptom queries respectively. At three symptoms, 99.1% of the possible combinations are returned within the top 10 results, and with four symptoms, all queries return COVID-19 within the top 10. These results highlight the speed with which a correct risk classification can be obtained, even when minimal symptoms are entered into the query.

**Figure 2:**
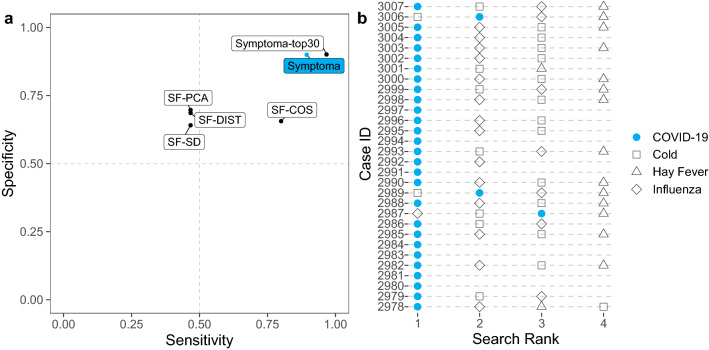
Performance by Symptoma and alternative approaches concerning the identification of COVID-19 cases. On the left, we show the performance of Symptoma, highlighted in blue, against alternatives predictors. All five are constrained to consider only three alternative diagnoses (the common cold, influenza, and hay fever) alongside COVID-19. We also give Symptoma’s accuracy on this set of case reports when unconstrained and evaluated as stated in the previous section (labelled as top30). On the right, we breakdown the predictions by Symptoma on the COVID-19 cases. Missing points indicate that the corresponding disease was considered so unlikely that it was not returned by the search.

### Symptoma performs better than simple approaches

Next, we compare Symptoma to alternative approaches used to predict the likelihood of bearing COVID-19. Using a restricted set of symptoms as input, the probability that a user is suffering from COVID-19 in comparison to either influenza, the common cold or hay fever is calculated based on their respective literature-derived symptom frequencies (see Table S1). We implemented four different methods: the distance in symptom-space between case presentation and symptom frequency (SF-DIST), the distance normalised by the standard deviation (SF-SD), the distance normalised by the first principal component (SF-PCA) and the cosine similarity (SF-COS). These methods are described in detail within the Methods.

To evaluate the performance of these approaches in comparison to Symptoma, we classified the combined COVID-19 and BMJ cases from Table 1, subsetting the later to only those cases that have at least one COVID-19 symptom. This gave a total of 404 cases (374 BMJ combined with 30 COVID-19). Please note that this is the same subset of cases used previously when evaluating Symptoma’s accuracy alone and is described in full within the Methods. A case is classified as COVID-19 positive if the probability of COVID-19 is higher than the probability for influenza, common cold or hay fever. As Symptoma weights COVID-19 against more than 20,000 other diseases, to provide a fair comparison, we note only the returned rank of influenza, the common cold and hay fever. If COVID-19 is returned first, we classify that case as COVID-19 positive.

The results, summarised in Fig. 2a, show that Symptoma performs considerably better than the alternative methods presented. A margin of 0.09 sensitivity and 0.20 specificity exists between Symptoma and any other method. This demonstrates the ability of Symptoma to differentiate between COVID-19 and other common causes with similar symptoms. Additionally, we show Symptoma’s performance using our previous measurement of accuracy outlined above (see Table 1) whereby the COVID-19 must be listed in the top 30 results.

In Fig. 2b, we breakdown Symptoma’s performance in the above test across our COVID-19 cases. We find that for the three cases that did not report COVID-19 as the preferred diagnosis, one is case 3006 outlined above as having a single defining feature of COVID-19. Case 2889, diagnosed as a common cold, symptoms include conjunctivitis and chills, two features more commonly associated with a cold. Lastly, case 2987, whose symptoms include nasal congestion and rhinorrhea, both indications of an alternative diagnosis to COVID-19.

### Symptoma userbase captures virus diffusion

Having validated Symptoma’s accuracy in identifying COVID-19, both with regards to general diagnoses and when only considering common diagnoses, we hypothesised that our self-administered online COVID-19 test powered by Symptoma would refect the overall levels of COVID-19 in a given population. To test this hypothesis, we compared the virus diffusion, taken as the new cases of COVID-19 reported daily, to the number of COVID-19 tests labelled as high-risk by Symptoma at a national level. This metric may be self-fulfilling, in that symptomatic people seek out our online test; however, this will be systematic and, therefore, trends over time can still be compared.

**Figure 3:**
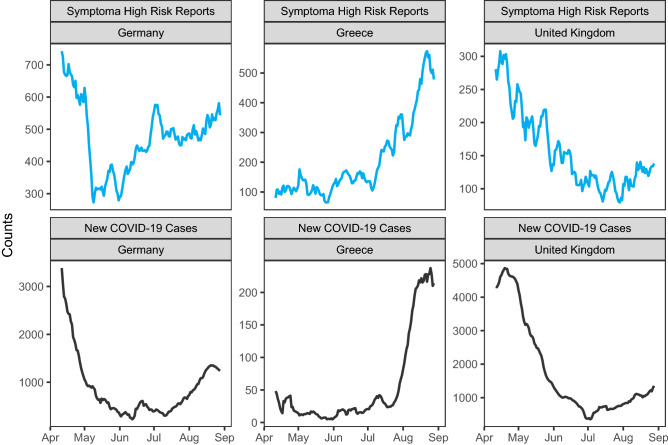
New cases of COVID-19 reported nationally compared to the number of COVID-19 online tests labelled as high-risk by Symptoma. Germany, Greece, and the United Kingdom are shown for the period 2020-04-11 to 2020-08-28. The Pearson correlation coefficient is 0.72, 0.91 and 0.92 respectively. Introducing a lag between these time series does not improve the correlation.

In Fig. 3, we show both metrics for Germany, Greece and the United Kingdom for the period 2020-04-11 to 2020-08-28. These are three of our top four countries by largest absolute userbases, with over 100,000 online tests with either high or medium risk for COVID-19 taken by each across the displayed period. Only the United States exceeds these national userbases, however, reported changes to their COVID-19 case reporting in May has led to wide questioning of their current figures^4–6^. For completeness, the figure for the US is given in the SI. We find, that for Germany, Greece and the United Kingdom, the trends are successfully encapsulated by Symptoma, with a Pearson correlation coefficient of 0.725 [0.64–0.80], 0.910 [0.88–0.93] and 0.925 [0.90–0.95] respectively.

To achieve such strong correlations, Symptoma captures both the initial reduction in new cases followed by second waves in both Germany and the United Kingdom. Likewise, for Greece, Symptoma reflects the excellent initial response, with few cases reported by either time-series, followed by a surge of cases beginning in August. Finding such strong correlations across our highest userbase countries is especially notable given it represents three different localisations of Symptoma (German, Greek, and English).

Lastly, we tested whether Symptoma was predictive or reactive with respect to these nationwide trends in new cases by introducing an offset between these time series. However, any offset in all three countries served only to reduce the correlation.

## Discussion

Above, we analysed the symptom-to-disease search engine Symptoma with regards to its diagnostic accuracy on cases of COVID-19. First, we showed that Symptoma can correctly identify cases of COVID-19, achieving an accuracy of 96.32% when considering more than 20,000 other potential causes. This far exceeds the 6,000 differential diagnoses provided by the second-largest symptom checker “Isabel Healthcare”^7^. Moreover, as Symptoma is constantly mining the newest literature, it keeps up-to-date with the latest knowledge and alters its predictions accordingly, e.g. reports of anosmia in COVID-19 patients followed the initial pandemic response^8,9^.

Secondly, we showed that Symptoma needs only a few symptoms to identify cases of COVID-19. Crucially, these can be inputted as free text that is semantically understood. For example, if one enters “Tiramisu”, “Food Poisoning” is returned as one of the top results. Moreover, Symptoma is localised into 36 languages, allowing a standardised approach to COVID-19 screening globally.

In contrast to Symptoma’s free-text capability, other COVID-19 questionnaires and symptom checkers typically require that patients only select from a predefined list of symptoms or are constrained to fixed questions (e.g., https://covid19.apple.com/screening/). As such, we showed that Symptoma performs better than these simple questionnaire-based approached in identifying COVID-19 cases over common misdiagnoses (influenza, common cold, and hay fever).

Lastly, we compared the number of COVID-19 assessments performed by Symptoma and found to be high-risk to the reported daily new cases in various countries. We showed that the correlation between these time series was up to 0.92, demonstrating that trends found by Symptoma correlate with the virus diffusion excellently. Given this, increases in the number of high-risk assessments made by Symptoma could be used to identify COVID-19 hotspots provided high enough user counts.

In summary, Symptoma provides a diagnostic accuracy with regards to COVID-19 higher than alternative approaches. It is usable in numerous languages and trends it exhibits correlates strongly with those found nationally. On the grounds, we believe that Symptoma is a highly valuable tool in the global COVID-19 crisis.

## Methods

### Symptoma

Symptoma uses (free-text) keywords and symptoms, age and sex as input from a user. The prediction engine then references a proprietary symptom-disease database curated by medical doctors and built upon a large text corpus that includes scientific publications, medical textbooks, patient self-reports, and Electronic Health Records, to map this input to possible causes. Alongside the database, predictions consider various factors, including, but not limited to, symptom occurrence frequency rates, country-specific disease incidences and feedback loops from specific user sessions. Symptoma’s algorithms rank over 20,000 causes and present the 30 most probable as suggested diagnoses to the user. Prior work describes the Symptoma engine and its diagnostic accuracy in more depth^3^.

### Test cases

To show the performance of Symptoma for COVID-19 we analysed a total of 1142 medical test cases. The different sets and sources of these cases are described below.

#### BMJ cases

A total of 1112 cases were sourced from the British Medical Journal (BMJ) and transcribed by a medical clinician into sets of symptoms, both negative and positive, alongside other risk factors, the patient’s age, and the patient’s sex when available^10,11^. The cases cover a diverse range of causes, including patients suffering rib fractures, rabies, or metastatic cancer. The number of symptoms and keywords per case ranges from 1 to 33 (median eight) including complex terms such as “right true vocal cord is immobile”.

A subset of the most pertinent cases was created by taking only those which included symptoms associated with either COVID-19 or diseases which are commonly mistaken for it. Namely, cases which reported any of the following symptoms: fatigue, dry cough, sneezing, malaise, rhinorrhea, sore throat, diarrhoea, headache, and dyspnea. All these symptoms, bar sneezing, are associated with COVID-19, as noted by the World Health Organisation (WHO)^12^. Sneezing is added to the symptom list due it being a known differentiator between COVID-19 and various other diseases with a similar presentation^13^. Under this constraint, 374 out of the 1112 BMJ cases were selected. Without sneezing, all cases would have still been selected. This subset represents cases with higher similarity to COVID-19 and, hence, an increased chance of misdiagnoses. Both common causes, such as influenza and the common cold, and rare causes, such as Avian influenza, are present within this subset.

#### Covid-19 cases

A set of 30 case reports were derived from the current literature, the sources of which are listed in full within the Supplementary Information. For each case, a list of symptoms and risk factors the patient presented with is given, alongside their age and sex where available.

#### COVID-19 cases: computer generated

We make use of our COVID-19 symptom list from above to construct example queries from those infected with COVID-19. These ten symptoms are combined with both “contact with someone infected with COVID-19” and “visiting/living in a COVID-19 risk area”, to give 12 possible symptoms and/or risk factors. All possible combinations of these are then taken as potential COVID-19 cases yielding a total number of 4096 artificial cases.

### Accuracy measurements

For any given set of symptoms, many possible causes could give rise to that specific presentation. We count a prediction as a true positive if the true cause is listed within the top 30 results returned by Symptoma. Note that this is the maximum number of causes returned by Symptoma for any given query. Given the possible 20,000 causes contained within Symptoma, this is the top 0.15%. Focussing on COVID-19, we can generate the following classification:

- True positive: COVID-19 case and COVID-19 returned in top 30 results.
- False positive: Non-COVID-19 case and COVID-19 returned in top 30 results.
- True negative: Non-COVID-19 case and COVID-19 not returned in top 30 results.
- False negative: COVID-19 case and COVID-19 not returned in top 30 results.

Throughout, we also assess a more strict threshold of COVID-19 being returned in the top 10 results. We refer to this stringent threshold as the “high-risk ” boundary.

### Alternative predictors

We developed four alternative methods to give the likelihood that a given patient has either COVID-19, influenza, common cold or hay fever. Underlying each were the frequencies with which various symptoms presented for these diseases (Table S1). To determine the probability of each disease, we represented each patient case in a 10-dimensional space, where each axis represents a different symptom. A value of one corresponds to exhibiting the symptom, zero means the patient does not have the symptom, and 0.5 means that they are unsure. This is fundamentally equivalent to many of the simple questionnaire-based approached to COVID-19 identification.

In the most simplistic approach (SF-DIST), we calculated the distance in space between the patient and each of the four diseases, each of which can also be seen as a point in the 10-dimensional symptom space. Normalisation yields the respective probabilities. In the second approach, the same procedure is used, but the distance in each dimension is scaled by the respective standard deviation of each symptom across all diseases (SF-SD). In the third approach, the distance in each dimension is scaled by the first principal component of a matrix consisting of all symptoms across all diseases (SF-PCA). Lastly, we interpreted the points as vectors and calculated the cosine similarity between the cases and diseases (SF-COS).

For assessing the accuracy of these approaches, we used the following criteria

- True positive: COVID-19 case and COVID-19 returned as most probable cause.
- False positive: Non-COVID-19 case and COVID-19 returned as most probable cause.
- True negative: Non-COVID-19 case and COVID-19 not returned as most probable cause.
- False negative: COVID-19 case and COVID-19 not returned as most probable cause.

Please note that we also assess Symptoma under similar criteria when comparing to these algorithms. Namely, we only consider the returned search rank of influenza, the common cold and hay fever. If COVID-19 is returned first, we classify that case as COVID-19 positive. Specifically, our definitions are the following

- True positive: COVID-19 case and COVID-19 returned first out of cause subset.
- False positive: Non-COVID-19 case and COVID-19 returned first out of cause subset.
- True negative: Non-COVID-19 case and COVID-19 not returned first out of cause subset.
- False negative: COVID-19 case and COVID-19 not returned first out of cause subset.

### Timeseries of new COVID-19 cases

To show that Symptoma captures the diffusion of COVID-19, we compare the new cases reported daily on a national level and the number of high-risk COVID-19 cases reported by our userbase. All counts were smoothed using a seven-day window box kernel to account for known fluctuations between weekday and weekend reporting. The Pearson’s correlation coefficient is used to give a measure of association and our stated confidence intervals are based on Fisher’s *Z* transform.

#### New COVID-19 cases nationally

For the number of new COVID-19 cases registered daily by various nations, we referenced the compiled statistics by Johns Hopkins University^14^.

#### High-risk COVID-19 tests

Since the start of the COVID-19 pandemic, Symptoma has offered free assessment as to one’s likelihood of being infected with COVID-19. This assessment prompts a user about known symptoms related to COVID-19 as well as allowing for free text input. Bar the prompted symptoms, this is no different from the regular usage of Symptoma. Here, we have analysed the number of these tests that return a high-risk for COVID-19 for various countries.

## Supplementary information

Supplementary Information.

## Data Availability

Results can be freely reproduced using the web interface https://www.symptoma.com.
